# Sonographic Evaluation of the Endotracheal Tube Position in the Neonatal Population: A Comprehensive Review and Meta-Analysis

**DOI:** 10.3389/fped.2022.886450

**Published:** 2022-06-02

**Authors:** Sabrina Congedi, Federica Savio, Maria Auciello, Sabrina Salvadori, Daniel Nardo, Luca Bonadies

**Affiliations:** ^1^Department of Woman's and Child's Health, University of Padova, Padua, Italy; ^2^Neonatal Intensive Care Unit, Department of Woman's and Child's Health, Padova University Hospital, Padua, Italy

**Keywords:** newborn, infant, neonate, endotracheal intubation, ultrasonography, POCUS

## Abstract

**Background:**

Endotracheal intubation in neonates is challenging and requires a high level of precision, due to narrow and short airways, especially in preterm newborns. The current gold standard for endotracheal tube (ETT) verification is chest X-ray (CXR); however, this method presents some limitations, such as ionizing radiation exposure and delayed in obtaining the radiographic images, that point of care ultrasound (POCUS) could overcome.

**Primary Objective:**

To evaluate ultrasound efficacy in determining ETT placement adequacy in preterm and term newborns.

**Secondary Objective:**

To compare the time required for ultrasound confirmation vs. time needed for other standard of care methods.

**Search Methods:**

A search in Medline, PubMed, Google Scholar and in the Cochrane Central Register of Controlled Trials (CENTRAL) was performed. Our most recent search was conducted in September 2021 including the following keywords: “newborn”, “infant”, “neonate”, “endotracheal intubation”, “endotracheal tube”, “ultrasonography”, “ultrasound”.

**Selection Criteria:**

We considered randomized and non-randomized controlled trials, prospective, retrospective and cross-sectional studies published after 2012, involving neonatal intensive care unit (NICU) patients needing intubation/intubated infants and evaluating POCUS efficacy and/or accuracy in detecting ETT position vs. a defined gold-standard method. Three review authors independently assessed the studies' quality and extracted data.

**Main Results:**

We identified 14 eligible studies including a total of 602 ETT evaluations in NICU or in the delivery room. In about 80% of cases the gold standard for ETT position verification was CXR. Ultrasound was able to identify the presence of ETT in 96.8% of the evaluations, with a pooled POCUS sensitivity of 93.44% (95% CI: 90.4–95.75%) in detecting an appropriately positioned ETT as assessed by CXR. Bedside ultrasound confirmation was also found to be significantly faster compared to obtaining a CXR.

**Conclusion:**

POCUS appears to be a fast and effective technique to identify correct endotracheal intubation in newborns. This review could add value and importance to the use of this promising technique.

## Introduction

In recent years point of care ultrasound (POCUS) has been shown to be an exciting tool in the neonatal field, not only helpful in the diagnosis and follow up of a large number of clinical conditions, such as respiratory distress syndrome, transient tachypnea of the newborn, pneumonia, atelectasis, pneumothorax (1, 2) bronchopulmonary dysplasia (3) but also useful in devices' positioning and monitoring (4), thus constituting a promising radiation-free aid in clinical practice (5). Some reports explored POCUS application in endotracheal tube (ETT) position's assessment although strong evidence is still lacking (4), as demonstrated by the presence only of single center small sized studies and the absence of randomized controlled trials and of a systematic review and metanalysis (6). Endotracheal intubation is a very common procedure both in neonatal intensive care units (NICUs) patients facing respiratory failure, and in emergency settings like neonatal resuscitation in the delivery room (7). Endotracheal intubation is challenging in neonates and requires a high level of precision, due to narrow and short airways, especially in preterm newborns. Confirmation of the correct ETT placement is mandatory, since its malpositioning is quite common. Indeed a selective bronchial intubation is reported in 7% of cases (8) and can lead to severe complications such as atelectasis, airleaks, with consequent inadequate ventilation and possibly leading to morbidities and even mortality (9, 10). On the other hand an ETT placed too high could lead to accidental extubation. Esophageal intubation has a reported incidence of 21.4% (8) but is usually promptly recognized. Moreover, in case of respiratory distress syndrome, incorrect ETT location will lead to ineffective or inadequate surfactant administration (11).

Chest X-ray (CXR), currently considered the gold standard, is the most frequently used method for ETT's position confirmation in newborns (12, 13). However, it presents some limitation, such as ionizing radiation exposure (14) especially when additional CXR is required (for example in case of unintended extubation or need for tube repositioning) (15). Furthermore, CXR is time consuming, particularly in emergency settings, such as in the delivery room or prior to surfactant administration (7) like during an InSurE procedure (Intubation—Surfactant administration—Extubation), which requires fast and temporary intubation. In this setting, given the impossibility of performing CXR, end tidal CO2 (EtCO2) monitoring was proposed to assess correct positioning of ETT (16).

So, up until now there is no evidence on which is the most effective method for ETT position assessment in neonates (14). The ideal technique should be fast, non-invasive, radiation free, reliable, easy to learn and perform. The simplest, but unfortunately imprecise, way may be the evaluation of clinical signs, such as prompt improvement of heart rate and oxygen saturation, presence of thoracic excursions, equal breath sounds bilaterally in the lungs, condensation in the ETT (17). Capnography is another method, based on exhaled CO2, but can lead to both false-negative results, for example in case of low cardiac output or too low inflation pressure in presence of severe respiratory failure, and false-positive results in the event of right mainstem intubation (18). EtCO2 monitoring is also recommended by the “European Resuscitation Council” to confirm endotracheal tube position during advanced life support in newborns, even if it does not permit identification of selective bronchial intubation (13). POCUS has already been demonstrated to be a reliable technique for endotracheal tube confirmation in adults (19, 20) and in the paediatric population (21) while evidence is lacking regarding neonatal patients (13). Children's unique thoracic anatomy provides many acoustic windows into the chest allowing a suitable thoracic POCUS examination. Moreover the incomplete thoracic ossification of the newborn warrants a better analysis of portions of the anterior thorax and mediastinum (22). There has recently been a growing interest in performing POCUS in the NICU's setting, thanks to its advantageous technical features such as non-invasiveness, absence of radiation exposure, bedside feasibility, fast execution and repeatability. Data available in literature provided some different methods for visualizing the ETT tip and determining its position in the newborn population. Dennington et al. suggested placing the probe on the suprasternal notch to provide a midsagittal plane and, in order to confirm the tip is being seen, the ETT could be moved in and out (23), while in other studies a direct visualization of the ETT in the trachea is preferred (24). Other authors searched for the “comet tail” artifact (25) or the “double line” as confirmation of the tube in the trachea, while the superior right portion of right pulmonary artery or the aortic arch are the anatomic markers used for suggest the corrected ETT position (26, 27).

## Objective

Primary objective: to evaluate POCUS capability in determining ETT placement adequacy in preterm and term newborns. Firstly, evaluating POCUS capability of identifying the presence of ETT in the trachea; then, its ability in detecting an appropriately positioned ETT as assessed by the gold standard technique. Secondary objective: to compare time required for POCUS confirmation vs. time needed for other standard of care methods (CXR and others).

## Methods

### Literature Searches

The study protocol was conducted in accordance with the Preferred Reporting Items for Systematic Reviews and Meta-Analyses (PRISMA) Statement.

A comprehensive search was conducted by three authors (SC, FS, MA) in Medline, PubMed, Google Scholar and the Cochrane Central Register of Controlled Trials (CENTRAL), from database creation to September 2021.

Manual searching of previous reviews and cross-references and contacting expert informants were undertaken to identify relevant papers to be analyzed. We also searched clinical trials registries for current and recently completed trials.

The search strategy included the following key words or Medical Subject Headings (MeSH) terms: “newborn”, “infant”, “neonate”, “endotracheal intubation”, “endotracheal tube”, “ultrasonography”, “ultrasound”; we adopted the Boolean operators “AND, OR” to connect our search words. We also used age filters by choosing the category “newborn” and considered papers published in the last 10 years (published after 2012).

### Inclusion and Exclusion Criteria

Included studies met the following criteria: (1) NICU population needing intubation/intubated infants; (2) evaluation of POCUS efficacy and/or accuracy in detecting ETT position vs. a defined gold-standard method; (3) randomized and non-randomized controlled trials, prospective, retrospective papers and cross-sectional studies published in peer reviewed journals after 2012 were considered.

We excluded those studies of which, despite our best effort, we were unable to obtain the full text, mixed neonatal and pediatric population, case reports and case series.

Data collection and analysis was performed using the recommendations of the Cochrane Neonatal Review Group (28). Two authors (SF, AM) screened independently each article, reviewing the titles and abstracts of the studies and excluded studies that did not meet the eligibility criteria. The three review authors (SF, AM, CS) subsequently examined the retrieved full text of eligible studies.

Three review authors (SF, AM, CS) independently assessed the methodological quality of eligible studies and extracted data using a data extraction form blank data sheets are available in the Supplementary Material. In the data extraction form we reported for each eligible study: first author, year of publication, country, study design, type of enrollment, sample size and features, blinding, primary and secondary objectives, gold-standard method reported, POCUS operators and equipment, principal results. In case of incomplete data from a published paper the authors (SF, AM, CS) contacted the corresponding author requesting further information. Discrepancy in data extraction was resolved by discussion and building a consensus.

### Quality Assessment and Risk of Bias

Quality assessment of each included study was evaluated independently by three review authors (SC, FS, MA) and cross-checked (FS for MA, MA for SC, SC for FS). Disagreements were resolved by discussion and consulting a fourth author (LB). Assessment of risk bias and applicability were performed using the QUADAS-2 tool (29), and the four key domains (patient selection, index test, reference standard, flow and timing) were investigated in each eligible study.

### Data Synthesis and Statistical Analysis

To describe and summarize the data extracted by the different studies, we used dedicated tables. Furthermore, for each study (whereas such data were available) POCUS sensitivity and specificity to detect ETT position according to the gold standard technique (CXR) were represented in the forest plot; cumulative sensitivity and specificity values were also calculated through the contingency table. A secondary sub analyses was then performed to analyze these features in the subgroups using the two most common anatomic markers.

### Statistical Software

RevMan software for Apple (5.4 version) was used for the statistical analysis including graphs of forest plot of sensibility and sensitivity and HRSOC curve. MedCalc.org was also used as an additional helpful tool.

## Results

### Study Selection

**Figure 1:**
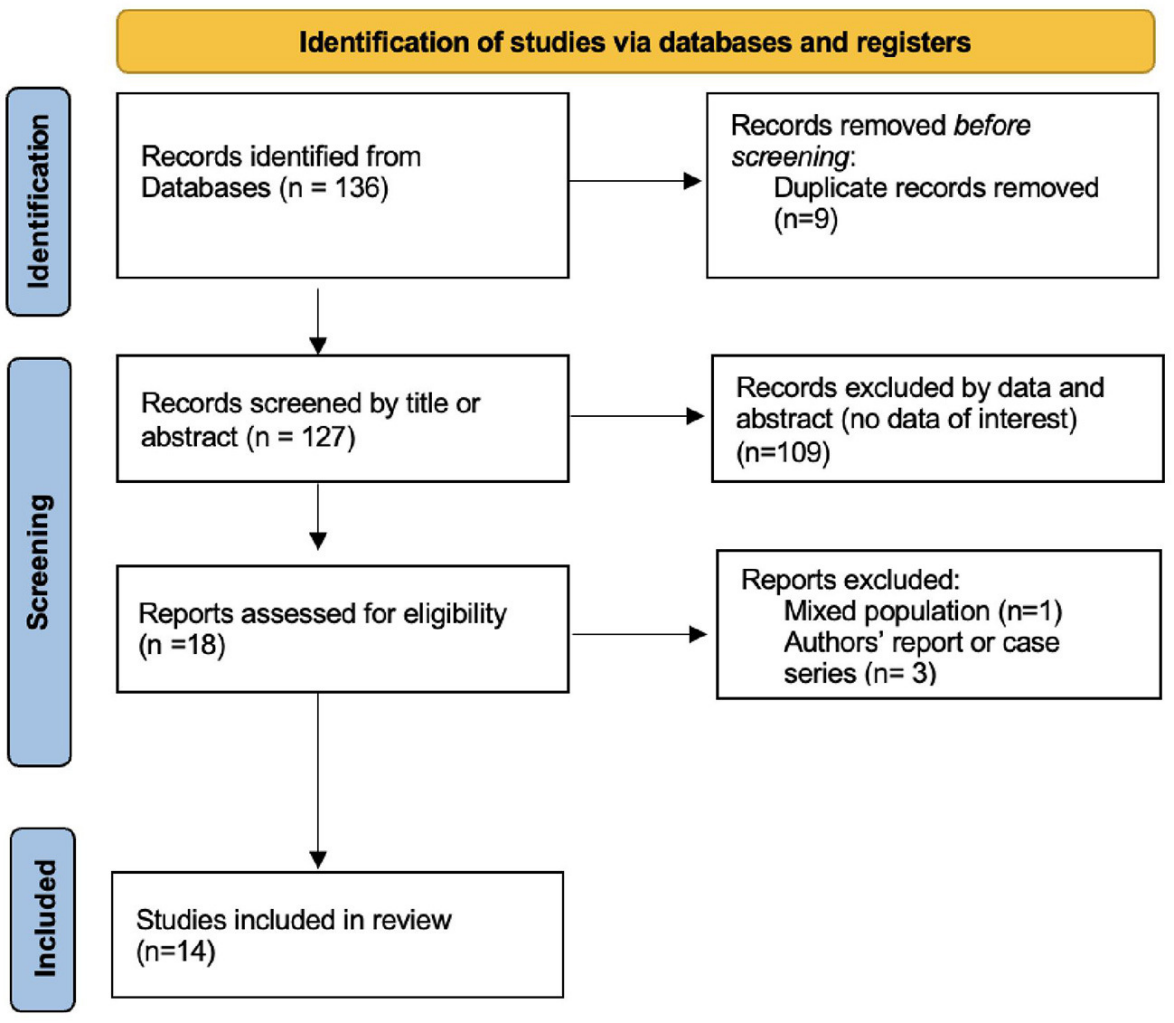
PRISMA flow chart study selection.

After a first screening based on title and abstract, and exclusion of duplicates, our electronic search for studies assessing the role of POCUS in the neonatal endotracheal intubation, provided a number of 18 studies. Full text of the studies was reviewed, and 4 other papers were excluded [1 due to mixed population and 3 were described as authors' experiences (30–33)]. Ultimately, 14 studies were included for data extraction and analysis. Flow chart of the search results, study selection log and included studies is presented in Figure 1, according to PRISMA guidelines (34).

### Study Characteristics

Following the above specified criteria we selected 14 studies, including a total of 602 ETT evaluation with a range from 9 to 143 procedures per work. The studies were published from 2012 to 2021 and conducted worldwide: 4 in the USA (23, 26, 35, 36), 5 in Europe [Spain (11, 25), Italy (37), France (24), Russia (38)], 4 in Asia [India (39, 40), Japan (41), Iran (27)], 1 in South Africa (42). Table 1 provides the details of the studies included in the analysis, while Table 2 shows the methods' summary. Regarding the 14 studies included, a consecutive patients' enrollment was realized only in 4 cases (35–37, 40). In the other cases, the sampling method was not well declared, but we could understand that patients were enrolled according to the availability of the sonographer. Sampling method was not consecutive but random, based on the staff work shifts. In Table 1 we reported the studies' level of evidence using the Oxford grading system (43).

**Table 1:** Details of the studies included in the analysis.

**References**	**Country**	**Study design**	**Sampling method**	**Oxford level of evidence**	**Sample size (NICU patients, *n*)**	**Gestational age, mean ±SD, wk**	**Birth weight,** **mean ±SD (range), g**	**ETT evaluations, when different from sample size (*n*)**
Alonso Quintela et al. (25)	Spain	Prospective	Unknown^*^	3	13	32 **±** 8	1,438, SD not available, (530–3,450)	
Chowdhry et al. (35)	USA	Prospective	Consecutive	3	29	28.3 **±** 4.7	1,282 **±** 866 (range not available)	56
De Kock et al. (42)	South Africa	Prospective	Unknown^*^	2	30	Unknown	1,600, SD not available, (1,200-−3,100)	
Dennington et al. (23)	USA	Prospective	Unknown^*^	3	28	30.2 **±** 4.9	1,595 **±** 862 (485–3,345)	29
Descamps et al. (24)	France	Not specified	Unknown^*^	3	30	30.8 **±** 5.1	1,612 **±** 1,086 (490–3,650)	52
Rodríguez-Fanjul et al. (11)	Spain	Prospective	Unknown^*^	3	12	33 **±** 1	1,384, SD not available, (1,100–2,150)	
Gorbunov et al. (38)	Russia	Prospective	Unknown^*^	3	42	29.7 **±** 5.2	Unknown	
Najib et al. (27)	Iran	Cross-sectional	Unknown^*^	2	40	Unknown	Unknown	
Salvadori et al. (37)	Italy	Prospective	Consecutive	2	71	28.9 **±** 5.4	1,272 ± 804.3 (630-1,900)	
Saul et al. (36)	USA	Prospective	Consecutive	3	9	Unknown	Unknown	
Sethi et al. (40)	India	Prospective	Consecutive	3	49	36.1 **±** 2.85	2,067 ± 653 (range not available)	53
Singh et al. (39)	India	Cross-sectional	Unknown^*^	2	143	30.8 **±** 4.6	Unknown	
Takeuchi et al. (41)	Japan	Retrospective	Unknown^*^	4	11	Mean not available, median 27 (23+5 to 30+4)	661, SD not available, (400–996)	12
Zaytseva et al. (26)	USA	Not specified	Unknown^*^	4	40	Unknown	Unknown	

*NICU, neonatal intensive care unit; n, number; wk, weeks; g, grams; SD, standard deviation; ETT, endotracheal tube*.

* *depending on sonographers' availability*.

**Table 2:** Methods summary of the included studies.

**References**	**Inclusion criteria**	**Exclusion criteria**	**Blinding**	**Objective**	**Gold standard**	**POCUS operators**	**POCUS method**	**POCUS equipment**
Alonso Quintela et al. (25)	NICU patients requiring intubation	not declared	Attending physician blinded to POCUS results	Efficacy of POCUS in assessing ETT correct placement	Capnography (intubation) CXR (ETT position)	1 Pediatrician (5 years' experience)	comet tail artifact (intubation), absence of acoustic shadow artifact in longitudinal scan (ETT position)	Vivid i, General Electrics, Atlanta, United States; 8 Hz microconvex array transducer and 12 Hz linear array transducer
Chowdhry et al. (35)	NICU patients requiring intubation	Congenital heart disease	No blinding	Efficacy of POCUS in assessing ETT position	CXR	POCUS technologists and pediatric radiologists	distance from the apex of the aortic arch	Phillips CX-50 Portable Ultrasound machine (Albany, NY, USA); curved 8–5 MHz transducer
De Kock et al. (42)	Intubated NICU patients	not declared	POCUS performed prior to CXR which was interpreted by a blinded radiologist	Efficacy of POCUS in assessing ETT correct placement	CXR	1 Radiologist	distance from the apex of the aortic arch	Toshiba, Nemio XG US machine; small curvilinear probe (6 MH)
Dennington et al. (23)	Intubated NICU patients	Upper airway anomalies	POCUS performer blinded to CXR (performed prior to POCUS)	Efficacy of POCUS in assessing ETT position	CXR	1 Neonatologist (expert), 1 Respiratory Therapist (trained by the previous one)	distance from the superior aspect of the right pulmonary artery	portable US machine (Vivid-i; General Electric Healthcare, Bethesda, Md., USA); high-frequency linear transducer (13 MHz)
Descamps et al. (24)	NICU patients requiring intubation or admitted intubated	Thoracic malformations	Neonatologists were blinded to each other and to CXR results	Efficacy of POCUS in assessing ETT correct placement	CXR	4 Neonatologists	distance from the apex of the aortic arch	Vivid S6 (General Electric Healthcare, Bethesda, Md., USA) device; 4–13 MHz transducer for visualization of the trachea; 5–11 MHz probe for verifying correct ETT position
Rodríguez-Fanjul et al. (11)	Newborns requiring surfactant administration	not declared	POCUS performer blinded to ETT placement depth and lung auscultation result	Usefulness of POCUS for confirmation of ETT placement during surfactant administration (InSurE protocol)	Lung auscultation	1 Neonatologist (with expertise in lung ultrasound)	ETT in the tracheal region and bilateral lung sliding for intubation	(Siemens Acuson X300, Siemens Healthcare GmbH, Erlangen,Germany); 10 MHz linear probe
Gorbunov et al. (38)	Intubated neonates	not declared	POCUS specialist and pediatric radiologist were blinded to the result of the other method	Efficacy of POCUS in assessing ETT correct placement	CXR	1 POCUS Specialist and 1 Pediatric Radiologist	distance from the apex of the aortic arch	Loqic S8 ultrasound machine; microconvex 4-10 MHz transducer
Najib et al. (27)	NICU patients requiring intubation	not declared	POCUS performer blinded to CXR and radiologist blinded to POCUS findings	Efficacy of POCUS in assessing ETT correct placement	CXR	1 Neonatologist (6 months trained)	distance from the superior aspect of the right pulmonary artery	Teknova TH-5100 portable US; 10 MHz linear probe
Salvadori et al. (37)	Intubated NICU patients who underwent CXR for any reason	Upper airway anomalies, diaphragmatic hernia, congenital heart disease	POCUS performer blinded to CXR results	Efficacy of POCUS in assessing ETT correct placement	CXR	2 Neonatologists (experts in functional echocardiography), 1 Pediatric resident	distance from the superior aspect of the right pulmonary artery	Vivid E9 echograph (GE Medical System, Milwaukee, WI, US); 11 MHz linear probe (for patients weighing > 1,500g); 12 MHz pediatric sector probe (for patients <1,500g)
Saul et al. (36)	NICU patients with ETT and radiographic confirmation within 24 hours	Lack of parental consent or patients clinically too unstable	Investigators blinded to CXR results	Efficacy of POCUS in assessing ETT and catheters position	CXR	1 Radiologist (30 years' experience in US), 1 Radiology resident (4 years' experience in US)	distance from the apex of the aortic arch	iU22 equipment (Philips Healthcare, Bothell, WA); linear 12–5-MHz transducer, supplemented by curved 8–5-MHz and linear 17–5-MHz transducers as needed
Sethi et al. (40)	NICU patients requiring intubation	not declared	Unknown	Efficacy of POCUS in assessing ETT correct placement	CXR	1 trained operator (2 weeks training)	distance from the apex of the aortic arch	Sonosite MicroMaxx portable US machine; 5–8 MHz probe
Singh et al. (39)	NICU patients requiring intubation	Tracheal, esophageal, cardiac, cranio-facial anomalies, generalized edema, low set ear, depressed nasal bridge	Investigators blinded to each other's POCUS findings	Normative data of the distance between ETT and anatomical structures across different weight and gestational age	CXR	2 trained operators	distance from the apex of the aortic arch	Sonosite M-Turbo portable ultrasound machine; 8–4 MHz phase array probe
Takeuchi et al. (41)	ELBW requiring intubation in delivery room	not declared	No blinding	Efficacy of POCUS in assessing ETT position vs. colorimetric method	Capnography	3 Neonatologists, 1 senior resident	comet tail artifact inside trachea for intubation	Fujifilm SonoSite M-turbo US device; body surface probe
Zaytseva et al. (26)	Neonates requiring oral intubation	Major congenital anomalies	Neonatologist blinded to the CXR measurements	Efficacy of POCUS in assessing ETT correct placement	CXR	1 Neonatologist	distance from the superior aspect of the right pulmonary artery	10 MHz cardiac probe (Zonare Z One PRO, Mindray, China)

*NICU, neonatal intensive care unit; ELBW, extremely low birth weight; ETT, endotracheal tube; POCUS, point of care ultrasound; US, ultrasound; CXR; chest X ray*.

Gestational age and birth weight of the newborns varied across the studies and were reported, where specified. 4 studies included extreme preterm infants <30 weeks (35, 37, 38, 41), 5 studies moderate preterm newborns 30-36 weeks (11, 23–25) and only one study (40) enrolled exclusively late preterm (>36 weeks). Mean birth weight of the patients included in the analysis, where clearly expressed, was >1,000 g, except in one case, where it was <1,000 g (41).

The inclusion criterion for the great majority of studies was need of intubation for any reason in patients admitted to NICU (23–27, 35–40, 42), with only two exceptions: in one case extremely low birth weight (ELBW) neonates were recruited when intubation was needed in the delivery room (41) and another one selected patients requiring surfactant therapy administered by InSurE technique (11).

Regarding exclusion criteria, when specified, patients presenting facial dysmorphism (26, 39) or upper airway or thoracic anomalies (23, 24, 26, 37, 39) were excluded for obvious reasons, as were patients with cardiac congenital defect (26, 35, 37, 39) since different position of aortic arch or right pulmonary artery would have interfered with ETT position estimation. Lack of parental consent was also considered (36).

The main objective of almost all the studies was to determine the efficacy of POCUS in assessing intubation and/or ETT position, in one case in relation to surfactant administration (11). Only one study had the specific goal of deriving normative data of the distance between ETT and anatomical structures across different weight and gestational age (39).

Regarding POCUS methods various markers have been proposed (Table 2). Images regarding probe position and ultrasonography projection are available in the Supplementary Material. When evaluating the mere tracheal intubation, 2 studies considered the comet tail artifact inside the trachea on cricoid transverse scan as marker of tracheal intubation (25, 41). On the other hand, when evaluating the precise position of ETT, the superior portion of the right pulmonary artery (RPA) was used as surrogate of carina anatomical position, while the apex of the aortic arch (AoA) was considered as a fixed point from which a distance of at least 0.5–1 cm was found to be appropriate. These POCUS scans were obtained from the suprasternal window with the probe placed in the midsagittal position. The distance between ETT tip and RPA was adopted by 4 studies (23, 26, 27, 37), while 7 studies measured AoA—ETT distance (24, 35, 36, 38–40, 42). Another study estimated the correct position assessing bilateral lung sliding and checking for the absence of acoustic shadow artifact in a longitudinal scan (corresponding to the air in the trachea if ETT was too high). For the other 2 studies the question about ETT exact position was not applicable (11, 41).

POCUS is performed by the figure of the neonatologist in half of the studies (11, 23, 24, 26, 27, 37, 41). Finally, POCUS equipment is quite heterogeneous in the different hospital centers of the studies included in our analysis, as showed in Table 2.

### Quality Assessment

**Figure 2:**
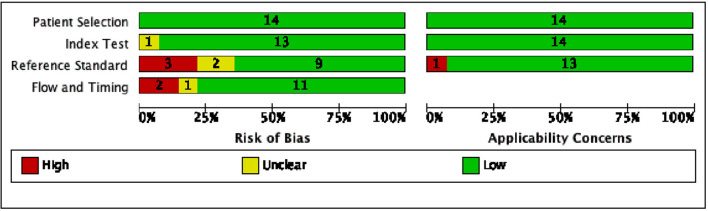
Risk of bias and applicability concerns graph according to QUADAS-2: review author's judgement about each domain presented as percentages across included studies.

**Figure 3:**
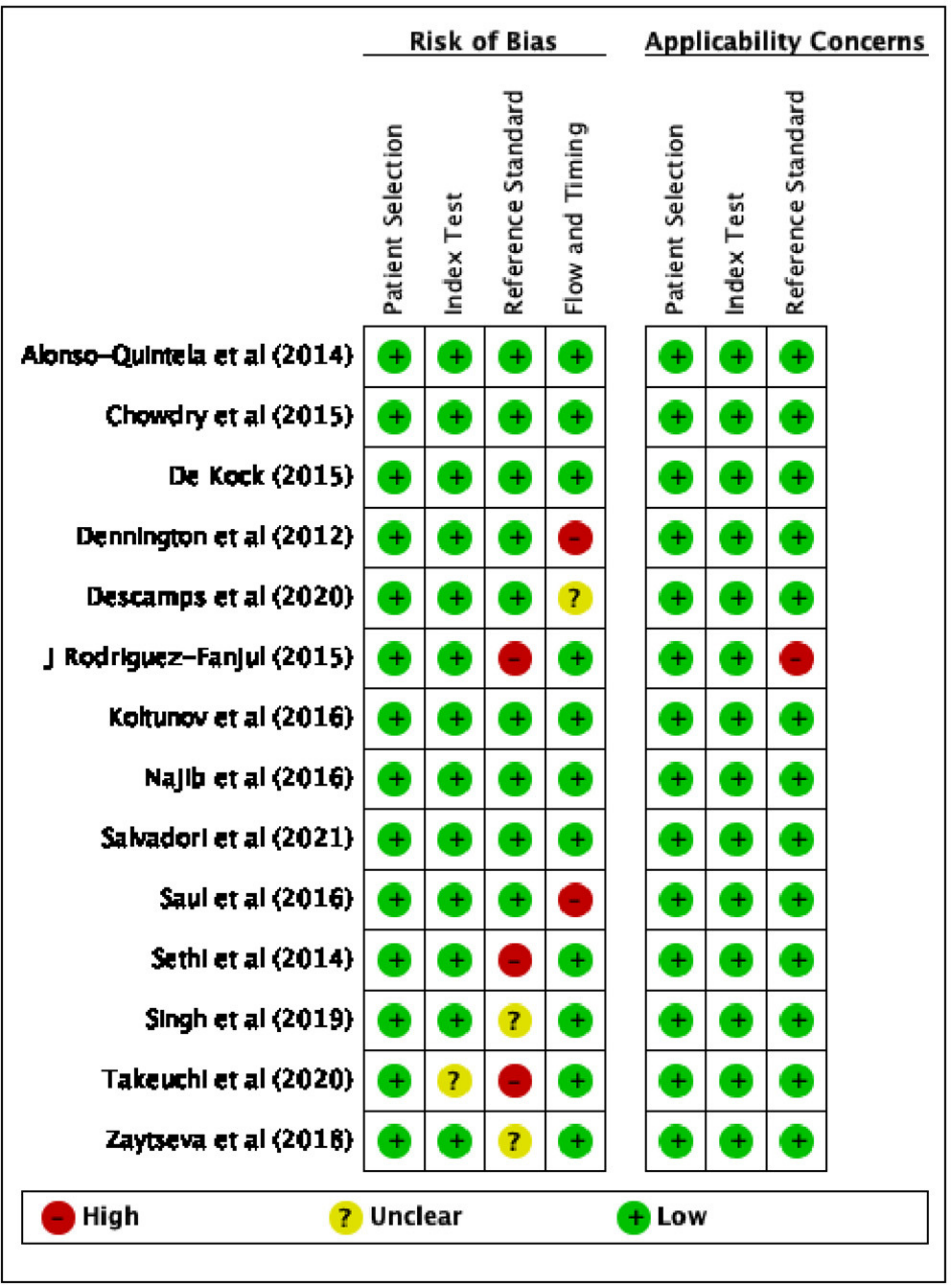
Risk of bias and applicability concerns summary according to QUADAS-2: review author's judgement about each domain for each included studies.

The results of the quality assessment, using QUADAS-2, of the risk of bias and applicability concerns of the selected studies are presented in Figures 2, 3.

Considering the patient selection domain, all studies were considered to carry low risk of bias. Regarding those studies that enrolled patients according to the availability of the sonographer, the sampling method was random, therefore we do not believe that this could be a source of bias.

As for the index test domain, all studies have been judged as having low risk of bias except one (35), since POCUS was performed simultaneously with the reference standard (capnography) and the operator might have known the result of both the exams.

With respect to the reference standard domain, 3 studies (11, 40, 41) were considered at high risk of bias and 2 studies (26, 39) at unclear risk because the reference exam was inadequate and the blinding status was not explicitly reported, respectively.

With regard to the flow and timing domain, 2 studies (23, 36) were considered as high risk of concern because several hours elapsed between POCUS and CXR, moreover in one study not all the tests were reported in the final analysis; only one study was described as not clear risk.

Regarding applicability, 1 study (11) was considered to be at high risk of bias in relation to the reference standard domain, since the auscultation is described as the reference exam. With respect to the index test and patients selection domains, all studies were considered to have low concerns.

### Role of POCUS in Detecting ETT in the Newborns

With respect to the data of the 14 selected studies, 602 ETT were investigated with POCUS. From the percentage numbers we derived data of interest (Table 3). In 583 cases (96.8%), POCUS identified the ETT.

**Table 3:** Number of intubation procedures described in the selected studies and POCUS efficacy in visualising ETT.

**References**	**ETT evaluations, n**	**POCUS efficacy in visualising ETT,** **% of cases**	**POCUS efficacy in visualising ETT, n of cases**
Alonso Quintela et al. (25)	13	85%	11
Chowdhry et al. (35)	56	100%	56
De Kock et al. (42)	30	100%	30
Dennington et al. (23)	29	100%	29
Descamps et al. (24)	52	96.1%	50
Rodríguez-Fanjul et al. (11)	12	100%	12
Gorbunov et al. (38)	42	100%	42
Najib et al. (27)	40	100%	40
Salvadori et al. (37)	71	100%	71
Saul et al. (36)	9	100%	9
Sethi et al. (40)	53	90.6%	48
Singh et al. (39)	143	93%	133
Takeuchi et al. (41)	12	100%	12
Zaytseva et al. (26)	40	100%	40
**Total ETT evaluations/Total ETT visualizing by POCUS** ***n*** **(% of cases)**			**583/602 (96.8%)**

*ETT, endotracheal tube, POCUS, point of care ultrasound*.

#### Accuracy of POCUS in ETT Placement in the Newborns

**Figure 4:**
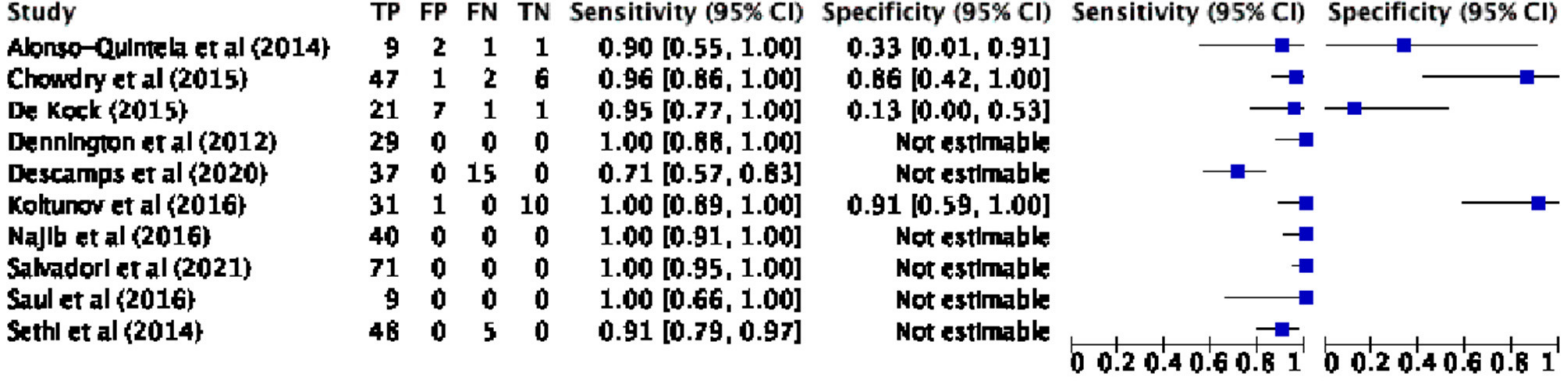
Pooled analysis forest plot: confirmation of ETT position by POCUS vs. CXR. ETT, endotracheal tube; POCUS, point of care ultrasound; CXR, chest X-ray; TP, true positive; FP, false positive; FN, false negative; TN, true negative; CI, confidence interval.

**Figure 5:**
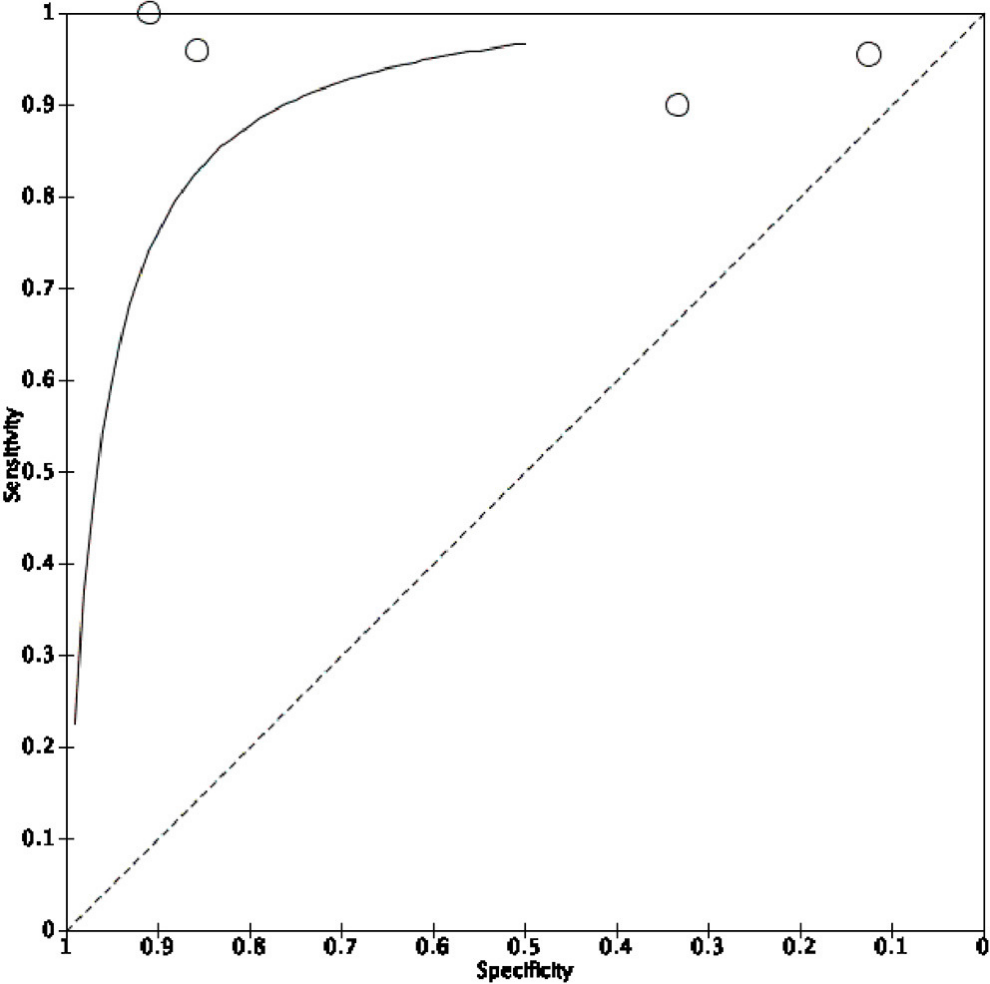
Summary of the pooled HSROC curve.

The diagnostic accuracy parameters (sensitivity, specificity, VPP, NPP) were derived by the results of the studies or, if not provided, from the raw numbers and vice versa using the Cochrane statistical package, RevMan V.5.4 software. Some studies were excluded from the accuracy analysis due to absence of specific data (26, 39) or different standard reference (e.g., capnography) (11, 41). Contingency table values, forest plot and HSROC curve regarding the pooled analysis are, respectively, reported in Table 4, Figures 4, 5.

**Table 4:** Pooled analysis contingency table.

		**Reference standard**		**Total**
		**+**	**–**	
**Index test**	**+**	True positive 342	False positive 11	**353**
	**–**	False negative 24	True negative 17	**41**
**Total**		**366**	**28**	**394**

The proportional metaanalysis revealed a pooled POCUS sensitivity of 93.44% (95% CI: 90.4–95.75%), intended as the POCUS ability in detecting an appropriately positioned ETT as assessed by the CXR. For those exams not capable of performing this task, the root cause was principally not sonography related, but due to malpositioned ETTs (ETT tip too high). In particular, Sethi et al. (40) were not able to detect ETT with POCUS in 5/48 patients, since the ETT tip was lying at or above the first thoracic vertebra; Najib et al. (27) could not see the ETT in 10/70 cases owing to it being higher than the thoracic inlet. On the other hand, few authors had difficulty in visualizing ETT by US due to poor quality of POCUS images: Singh et al. (39) in 10/143 patients; Descamps et al. (24) in 2/52. Moreover, one study (26) was excluded from the analysis, due to the absence of the data; authors were contacted but no response was obtained.

POCUS specificity was offered only by few studies because of the lack of true negative (TN) values (i.e., paucity of malpositioned ETT identified with the gold standard), the pooled POCUS specificity was 60.71% (95% CI: 40.58–78.5%). We obtained a PPV of 96.88% (95% CI: 95.15–98.01%) and NPV of 41,46% (95% CI: 30.35–53.58%).

We conducted a subevaluation regarding capability of POCUS in detecting a comparable ETT distance from carina compared with CXR. As regards the measurement of the distance between ETT and the superior aspect of the right pulmonary artery by POCUS, the data of 3 studies (23, 27, 37) can be extrapolated from the Bland Altman plot. If we consider +/– 0.75 cm as an acceptable deviation measure with respect to the depth of the ETT measured with the CXR, 93% of the measurements were in agreement. As arbitrary as the 0.75 cm value is, it appears to be a reasonable value from a clinical point of view, as repositioning the ETT is not a risk-free process.

We also tested separately the two mostly used anatomic markers “distance from the superior aspect of the right pulmonary artery” and “distance from the aortic arch apex. The first showed a sensitivity of 100% (95% CI: 97.4–100%), whereas specificity was not calculable following the absence of any true negative and false positive results. The latter showed a sensitivity of 89.35% (95% CI: 84.45–93.13%) and a specificity of 65.38% (95% CI: 44.33–82.79%).

#### Time Needed to Perform POCUS to Confirm ETT Position in the Newborns vs. the Reference Exams

**Figure 6:**
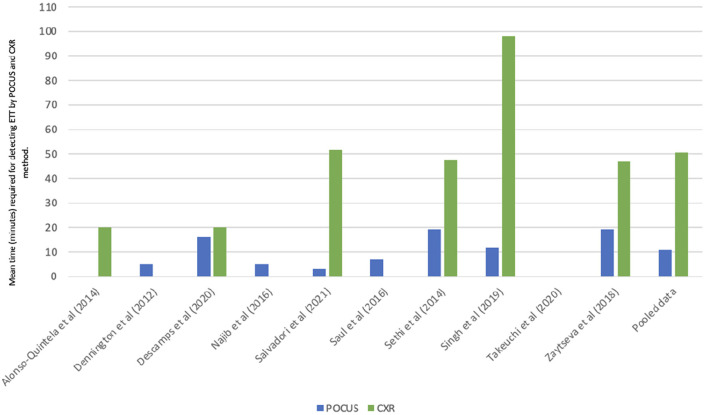
Mean time (minutes) required for ETT confirmation by POCUS, compared with CXR (when performed and time information available). ETT, endotracheal tube; POCUS, point of care ultrasound; CXR, chest X-ray.

Some of the studies selected in our research described the mean time needed to perform POCUS exams, with a variable range from seconds to minutes (Table 5 and Figure 6). A weighted average for the POCUS exams of about 10 min and 39 s can be described. Finally, some of these studies have also reported the time needed to perform the reference exams (CXR, Figure 6, or, in two cases, capnography). A weighted average to perform CXR can be estimated in 51 min.

**Table 5:** Time required for ETT position confirmation by POCUS and the reference exam.

**References**	**Time to perform POCUS, mean ± SD (range), minutes**	**Time to perform CXR, mean ± SD (range), minutes**	**Time to perform capnography, mean ± SD (range), seconds**
Alonso Quintela et al. (25)	0.21, SD not available, (0.17–0.40)	20, SD not available, (17–25)	6, SD not available, (3–12)
Chowdhry et al. (35)	Unknown	Unknown	
De Kock et al. (42)	Unknown	Unknown	
Dennington et al. (23)	<5 min, SD and range not available	Unknown	
Descamps et al. (24)	16 ± 9.5, range not available	20 ± 6.6, range not available	
Rodríguez-Fanjul et al. (11)	Unknown	Unknown	
Gorbunov et al. (38)	Unknown	Unknown	
Najib et al. (27)	<5 min, SD and range not available	Unknown	
Salvadori et al. (37)	3.2 ± 2.5, (1–13)	51.7 ± 40.7, (5–228)	
Saul et al. (36)	7, SD and range not available	Unknown	
Sethi et al. (40)	19.3 ± 7.9, range not available	47.3 ± 9.0, range not available	
Singh et al. (39)	Median 12 (8–15)	Median 98 (64–132)	
Takeuchi et al. (41)	Median 3 seconds, range not available	Not applicable	Median 11 s, range not available
Zaytseva et al. (26)	19.3, SD and range not available	47, SD and range not available	

*ETT, endotracheal tube, POCUS, point of care ultrasound, CXR, chest X-ray, SD, standard deviation*.

## Discussion

This meta-analysis was novel in evaluating the accuracy and diagnostic value of POCUS for detecting ETT and assessing its placement. Previous meta-analysis studies described the diagnostic performance of POCUS regarding transient tachypnea of the newborn (TTN) (44, 45), respiratory distress syndrome (RDS) (46) and bronchopulmonary dysplasia (BPD) (47). The pooled results obtained from the selected studies demonstrated that POCUS can recognize ETT in 96.8% of cases and describe its correct placement with a pooled sensitivity of 93.44% ranging from 0.71 to 1.00.

Our comparison between POCUS and the standard technique (CXR) for the evidence of ETT and its correct placement in the neonatal population demonstrates that ultrasound examination has a good sensitivity. The findings of this meta-analysis confirm POCUS high diagnostic value concerning both items. Furthermore, the distance of ETT from the superior aspect of the RPA showed a higher sensitivity when compared with the distance from the AoA, whereas specificity was not calculable for the first marker. We believe that POCUS markers, intended as distance between ETT and RPA or between ETT and AoA, should be evaluated together in a multicentric study in order to determine the most accurate, fast and easy to perform the technique.

All the studies that considered radiography as the gold standard have shown that ultrasound is faster than radiography in confirming intubation and assessing the position of the ETT. However, some works analyzed the time needed for the ultrasound evaluation (since POCUS equipment was already available at the patient's crib) vs. the time needed for CXR to be performed, while others evaluated the activation time of the ultrasound service compared to the radiographic one from the moment of intubation. Therefore, when POCUS equipment was ready, ultrasound investigation proved to be very fast, from an average of 3 s [according to Takeuchi's work (41)] to <5 min in the other studies.

Regarding the assessment with capnography, this was faster compared to POCUS in one study (41) but slower in a second study (25). Possibly this difference can be justified by the population studied; indeed, the second study (25) considered only ELBW infants, a specific population where capnography can lead to false negative results or positivity latency in relation to low lung volumes (13).

It is important to consider several limitations with respect to the present meta-analysis. Patients' enrollment could not be consecutive in the great majority of the included studies since POCUS performers were not available 24/24 h and 7/7 days. Furthermore, personal experience with POCUS examination certainly plays a notable role in accuracy and speed of the echographic evaluation that we could not take into account in our analysis (40).

The 14 studies presented a low rate of ETT malposition (too high or too low inside the trachea) and an even lower rate of esophageal intubation, the latter probably evident to clinical evaluation, thus POCUS specificity in detecting ETT tip correct position is not reliable. Importantly, we must consider that a great number of ETT evaluations were not done immediately after the intubation procedure, but in already intubated and mechanically ventilated infants seeking to confirm the ETT (presumably adequately placed) and compare POCUS with CXR. In other cases (27, 39), POCUS was performed exclusively in well positioned ETT. We acknowledge that POCUS has some concerns regarding specificity but, considering the high sensibility and the high PPV, the impossibility of visualization of the ETT through this method could suggest a position that is too high. In this circumstance, probably, trying to deepen the ETT under ultrasound guidance could help.

Finally, we were not able to collect all data from all the studies: given the heterogeneity of the studies included in this meta-analysis, we tried to formulate homogeneous questions by requesting further data from the authors but we did not get all the answers.

## Conclusions

One of the main challenges is to offer stronger evidence to promote the changing of clinical practice. Our meta-analysis shows that POCUS appears to be a fast and effective technique for identifying the appropriateness of ETT position in the NICU population.

More studies are needed in order to establish POCUS specificity, a large trial of POCUS performed immediately after intubation could offer a higher rate of malpositioned ETT and hence be more informative about specificity. POCUS training in ETT visualization in the newborn and comparison between different anatomical markers are topics worthy of further studies.

## Author Contributions

SC, FS, MA, and LB: conceptualization, reviewing literature, drafting, writing, critically editing for important intellectual content, final approval of the version to be published, and agreeing to be accountable for all aspects of the work. SC, FS, and MA: reviewing literature, drafting, conducting statistical analysis, co-writing of the work. LB: solved questions related to the accuracy or integrity of any part of the work, reviewed the first draft of the paper. LB, SS, and DN: critically editing for important intellectual content, final approval of the version to be published. All authors contributed to the article and approved the submitted version.

## Conflict of Interest

The authors declare that the research was conducted in the absence of any commercial or financial relationships that could be construed as a potential conflict of interest.

## Publisher's Note

All claims expressed in this article are solely those of the authors and do not necessarily represent those of their affiliated organizations, or those of the publisher, the editors and the reviewers. Any product that may be evaluated in this article, or claim that may be made by its manufacturer, is not guaranteed or endorsed by the publisher.
