# Supplementary material for: Sonographic Evaluation of the Endotracheal Tube Position in the Neonatal Population: A Comprehensive Review and Meta-Analysis
Source: Front Pediatr. 2022 Jun 2;10:886450. doi: 10.3389/fped.2022.886450 (PMC9201277; doi:10.3389/fped.2022.886450)
Supplement: Supplementary file 1 [file Data_Sheet_1.DOCX]

| Author, year | Country | Study design | Sampling method | Oxford level of evidence | Sample size  (NICU patients, n) | Gestational age, mean ± SD, wk | Birth weight,  mean ± SD (range), g | ETT evaluations, when different from sample size (n) | Inclusion criteria | Exclusion criteria | Blinding | Objective | Gold standard | POCUS operator | POCUS Method | POCUS equipment | ETT visualized by POCUS n (%) | POCUS sensibility | POCUS specificity | POCUS performing time (mean + sd) | Gold standard performing time (mean + sd) |
| --- | --- | --- | --- | --- | --- | --- | --- | --- | --- | --- | --- | --- | --- | --- | --- | --- | --- | --- | --- | --- | --- |
|  |  |  |  |  |  |  |  |  |  |  |  |  |  |  |  |  |  |  |  |  |  |
